# Undetected Small Accessory Mental Foramina Using Cone-Beam Computed Tomography

**DOI:** 10.7759/cureus.1210

**Published:** 2017-05-02

**Authors:** Joe Iwanaga, Koichi Watanabe, Tsuyoshi Saga, Shogo Kikuta, Yoko Tabira, Sadaharu Kitashima, Christian Fisahn, Fernando Alonso, R. Shane Tubbs, Jingo Kusukawa, Koh-ichi Yamaki

**Affiliations:** 1 Seattle Science Foundation; 2 Department of Anatomy, Kurume University School of Medicine; 3 Dental and Oral Medical Center, Kurume University School of Medicine; 4 Kitashima Dental and Orthodontic Clinic; 5 Orthopedic Surgery, Swedish Neuroscience Institute; 6 Neurosurgery, University Hospitals of Cleveland, Case Medical Center; 7 Neurosurgery, Seattle Science Foundation

**Keywords:** cone-beam computed tomography, anatomic variation, mandible, three-dimensional, oral surgical procedures, anatomy

## Abstract

**Introduction:**

The accessory foramina could not be identified on some imaging modalities such as surface-rendered images. The purpose of this study was to investigate the ability of surface-rendered images in detecting these foramina.

**Materials and methods:**

We analyzed 20 accessory mental foramina (AMF) in nine mandibles removed from cadavers with cone-beam computed tomography (CBCT) and assessed in surface-rendered images. All AMF were divided into three groups depending on their visibility.

**Results:**

Group 1 included AMF that were clearly visible as foramina, Group 2 were not clearly visible but could be recognized with concave parts, and Group 3 were not visible and the smooth surface of the bone was observed. Group 1 ranged from 1.3 to 5.1 mm^2^, Group 2 from 0.3 to 3.8 mm^2^, and Group 3 from 0.2 to 1.1 mm^2^. A statistically significant difference in the mean size between Groups 1 and 3 was observed. Even if the AMF are smaller (e.g., 1 mm in diameter), they should still be avoided to prevent injury.

**Conclusions:**

The clinician should be aware that smaller foramina might not be detected on these images.

## Introduction

Accessory mental foramina (AMF) are an anatomic variation of the mandible. The incidence of AMF ranges from 2.0% [1] to 14.3% [2]. Dentists and oral surgeons have found AMF during oral procedures including implant surgeries [3], periapical surgeries [4-5], trauma surgeries [6-7], and neurectomies [8-9]. However, it is very difficult to find AMF using only panoramic images [10-11], and their existence can go unnoticed by clinicians until after the periosteum is elevated. With the development of cone-beam computed tomography (CBCT), we are now able to diagnose AMF more easily. Details (e.g., location, size, number, relationship with the mandibular canal) of AMF previously only available with non-clinical research methods can now be identified with clinical CBCT. As with any imaging modality (including CBCT), the image resolution limits the detectability of anatomical features. Although there are manufacturing differences between CBCT systems, the images obtained can generally be displayed as volume-rendered, surface-rendered, or three-dimensional (3D) images. During the development of a treatment plan, especially for implant surgery, many dentists work from volume and surface-rendered images after verifying the position of the mental foramen on the cross-sectional images. In our previous study, we found that the detection of AMF was dependent on the image type [2]. These foramina could not be identified on some imaging modalities such as surface-rendered images. As a result, some AMF may go undetected if overlooked on cross-sectional images. Arx [5] described that nerve paralysis had occurred after cutting the accessory mental nerve which came out from the large accessory mental foramen. So detecting the accessory mental foramina is very important for surgeons to avoid injuring accessory mental nerves. To our knowledge, there have been no studies describing this lack of detection. Therefore, the purpose of this study was to investigate the ability of surface-rendered images in detecting these foramina.

## Materials and methods

In our previous study [2], 20 AMF on nine adult cadaveric mandibles were examined with CBCT (GALILEOSR, Sirona, Germany) (image acquisition parameters: 85 kV, 6 mA). Axial images were transmitted in the digital imaging and communication in medicine (DICOM) format, and two-dimensional images of the body of the mandible were reconstructed using the OsiriX DICOM viewer (Pixmeo, Geneva, Switzerland) [12]. Each AMF size and location was determined and additional features were identified in the 3D reconstructed images. The area of each foramen was calculated using the following formula: elliptical area = π x (long axis)/2 x (short axis)/2. The lengths of the long and short axes, as calculated from the CBCT images, were assumed to represent the physical values as no difference in length was previously observed when comparing the two methods [2].

**Figure 1:**
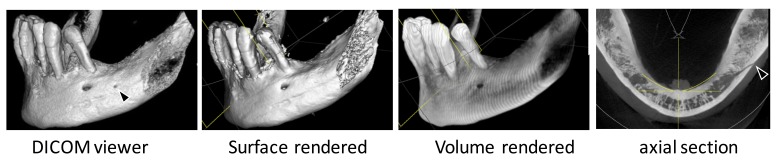
One accessory mental foramen was located posterior to the mental foramen as seen with the DICOM viewer and axial section (black arrowhead) but no accessory mental foramen could be recognized on surface- and volume-rendered images.

In this study, the same 20 AMF were assessed on surface-rendered images, which are often used in daily dental practice. The volume-rendered images were excluded because almost all of the AMF were not detectable on them (Figure 1).

**Figure 2:**
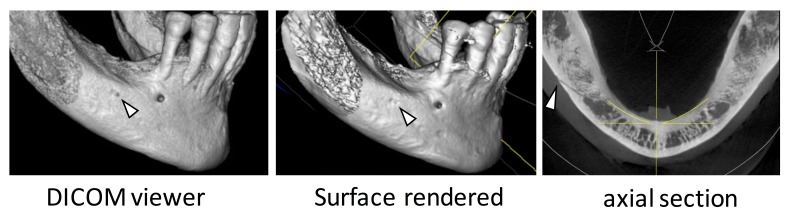
One accessory mental foramen (white arrowhead) located posterior to the mental foramen was seen with the DICOM viewer but only the concave part could be seen on the surface-rendered image.

**Figure 3:**
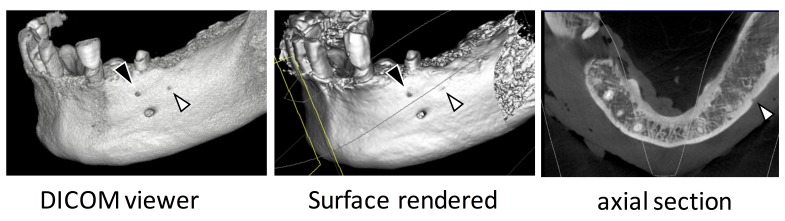
One accessory mental foramen (black arrowhead) was identified with the DICOM viewer and seen on the surface-rendered and axial images. The other accessory mental foramen (white arrowhead) was seen as a concave feature on the surface-rendered image.

All AMF were reviewed in the surface-rendered images and divided into three groups depending on their visibility. The initial image window/level setting was not changed and the threshold value was set at 1600 HU. Group 1 included AMF that were clearly visible as foramina, Group 2 were not clearly visible but could be recognized with concave parts (Figure 2-3), and Group 3 were not visible and the smooth surface of the bone was observed (Figure 1).

The differences between the groups were evaluated using a paired t-test with a p-value < 0.05 considered as significant. In this study, the AMF were defined as a smaller foramen compared with the mental foramen (MF), which had continuity with the mandibular canal [13]. As a cadaveric examination, the present study did not require approval by an ethics committee at our institutions and the work was performed in accordance with the requirements of the Declaration of Helsinki (64th WMA General Assembly, Fortaleza, Brazil, October 2013).

## Results

The size and visibility of all 20 AMF are shown in Table 1. Group 1 ranged from 1.3 to 5.1 mm², Group 2 from 0.3 to 3.8 mm², and Group 3 from 0.2 to 1.1 mm². The larger AMFs, which ranged in size from 1.3 to 5.1 mm², were classified as Groups 1 or 2. The smaller AMFs, which ranged in size from 0.2 to 1.2 mm², were classified as Groups 2 or 3 (Table 1).

**Table 1:** The size and visibility of all 20 AMF

Size (mm^2^)	Group 1	Group 2	Group 3
0.2			+
0.2			+
0.3		+	
0.4		+	
0.6			+
0.9			+
1.1			+
1.1		+	
1.1			+
1.1		+	
1.2		+	
1.3	+		
2.1	+		
2.7		+	
2.9		+	
3.1	+		
3.2	+		
3.4	+		
3.8		+	
5.1	+		

. The range, mean, and median for the AMF are shown in Table 2.

**Table 2:** The range, mean, and median for the AMF

	Range (mm^2^)	Mean ± SD (mm^2^)	Median (mm^2^)
Group 1	1.3 - 5.1	3.03 ± 1.29	2.7
Group 2	0.3 - 3.8	1.69 ± 1.28	1.2
Group 3	0.2 - 1.1	0.68 ± 0.42	1.0

A statistically significant difference in the mean size between Groups 1 and 3 (p < 0.05) was observed, while no statistically significant differences between Groups 1 and 3 or Groups 2 and 3 (p > 0.05) were observed (Table 3).

**Table 3:** Difference in the mean size

	p value	Significance
Group 1 and 2	0.066	not significant
Group 1 and 3	0.004	significant
Group 2 and 3	0.289	not significant

.

## Discussion

In clinical practice, CBCT images are often utilized for diagnosis before oral surgery. The use of CBCT has increased, and approximately 10% of dental clinics in Japan have a dedicated CBCT system [14]. Although the guidelines for clinical use [15], optimal scanning protocol [16], and the relationship between the Hounsfield units and thickness of cortical bone [17] have already been reported, it is well known that CT image characteristics change depending on the applied threshold of CT numbers. There have been no reports that have compared AMF on surface-rendered images (often used by general dental practitioners) with AMF on 3D-CT images using a DICOM viewer (typically more realistic). In addition, oral surgeons and oral radiologists are familiar with interpreting CT images of the mandible, while many general dentists are not. Most previous reports regarding AMF have been authored by oral surgeons, radiologists, or anatomists [2, 13, 18]. These studies have analyzed AMFs in both cross-sectional and 3D reconstructed images, but not in surface rendered images, which many general dentists use for developing treatment plans. Unfortunately, general dentists are not very familiar with the term AMF although these can be clinically important [1-2, 18].

In the present study, a significant difference was seen between Groups 1 and 3, indicating that visible AMF were larger than those not visible. However, all AMF smaller than 1.3 mm² were not clearly identified and might have the potential to be overlooked and thus injured during surgery. As Wang, et al. [19] described, a large bony canal (> 1 mm in diameter) should be identified before surgery in order to avoid hemorrhage. Even if the AMF are smaller (e.g., 1 mm in diameter), they should still be avoided to prevent injury.

We suggest two important changes to practice in order to avoid hemorrhage or other complications during surgery. The first is to educate dentists on the existence and characteristics of AMF. The second is to ensure that clinicians review CT images knowing that AMF may exist and what their imaging characteristics are.

There are several limitations in this study. First, any statistical differences between foramina that included nerves and/or arteries were not taken into consideration because there were only three of these. Secondly, the location and number of AMF were known before creating the surface-rendered images. If the authors did not know the location and number of AMF on the 3D images, some of the AMF in Group 2 may have been incorrectly classified as Group 3 AMF. Thirdly, differences in the physical position of the AMF were not taken into account. The X-ray beam direction and cortical bone thickness may have varied between patients and might have affected visibility. Finally, due to the inherent manufacturing differences between CBCT scanners, image acquisition parameters, and resolution, other studies might observe different results.

## Conclusions

In this study, 20 AMF were assessed using CBCT and the AMF smaller than 1.3 mm² were not clearly identified on surface-rendered images. Clinical anatomists should let general dentists know this important knowledge about clinical practice for dentistry.
